# Correlations between environmental salinity levels, blood biochemistry parameters, and steroid hormones in wild juvenile American alligators (*Alligator mississippiensis*)

**DOI:** 10.1038/s41598-021-94557-y

**Published:** 2021-07-26

**Authors:** Patricia C. Faulkner, Ruth M. Elsey, David Hala, Lene H. Petersen

**Affiliations:** 1 Department of Marine Biology, Texas A&M University At Galveston, Galveston Campus, 200 Seawolf Parkway, Bldg. 3029, Galveston, TX 77553 USA; 2 Louisiana Department of Wildlife and Fisheries, Rockefeller Wildlife Refuge, Grand Chenier, LA 70643 USA

**Keywords:** Physiology, Environmental sciences, Endocrinology

## Abstract

American alligators (*Alligator mississippiensis*) inhabit freshwater wetlands that are vulnerable to salinization caused by anthropogenic alterations to freshwater flow, in addition to storm surges, sea level rise, and droughts. Salinization of coastal freshwater habitats is a growing concern in a changing climate due to increased frequency and intensity of storm surges and drought conditions. This study opportunistically sampled juvenile male and female wild alligators in various salinities each month excluding November, December, and January for one year at Rockefeller Wildlife Refuge in coastal Louisiana. Blood plasma biochemistry parameters including electrolyte levels were subsequently measured. In addition, levels of various renin–angiotensin–aldosterone system hormones, glucocorticoids, androgens, estrogens, and progestogens were analyzed using liquid chromatography and tandem mass spectrometry. Only males were sampled in hyperosmotic environments (> 10‰) during dry conditions in late summer 2018. In juvenile males, plasma Na^+^, Cl^−^, and the progestogen 17α,20β-dihydroxypregnenone were significantly and positively correlated with environmental salinity. However, variation in glucocorticoids, androgens, and estrogens were not associated with hypersaline water while sex steroids showed significant seasonal variation. This study demonstrated significant correlation of environmental salinity with electrolyte levels and a sex steroid in wild juvenile alligators, and to our knowledge represents the first measurement of 17α,20β-dihydroxypregnenone in alligators.

## Introduction

American alligators (*Alligator mississippiensis*)^1^ do not tolerate saline environments for prolonged periods of time without access to freshwater^2–5^. However, many freshwater alligator habitats are prone to frequent seawater intrusion from storm surges^6,7^. Rivers, lakes, and freshwater wetlands are frequently exposed to salinization due to anthropogenic perturbations that cause changes in freshwater or underground water flow^8,9^. Furthermore, gradually rising sea levels may introduce saline water into inland groundwater fed wetlands resulting in salinization of inland areas not directly affected by seawater intrusion^10^.

Juvenile alligators are especially at risk of salinization effects due to their smaller size and thinner skin, allowing more rapid loss of water^11,12^. Since American alligators are a keystone species that control populations at lower trophic levels, salinization events can have disruptive ecosystem-level impacts^13,14^. Salinization is a growing concern for coastal freshwater habitats due to the effects of climate change. For instance, projected increases in sea surface temperature are associated with growing intensity and frequency of hurricanes with ensuing storm surges^7,15,16^. Furthermore, frequency of drought conditions is projected to increase due to rising greenhouse gas emissions^17,18^, contributing to increased salinity in wetlands and exacerbating salt inundation as seen in southwest Louisiana following Hurricane Rita^11^.

Alligators use various osmoregulatory mechanisms to cope with changing salinity levels. For instance, they can modify the composition of urine in the kidney to excrete higher levels of sodium and chloride^3,4^, although they are unable to excrete urine hyperosmotic to body fluids due to their kidney nephrons lacking loops of Henle^19^. In addition, alligators are known to behaviorally osmoregulate by selecting lower-salinity habitats^12,20,21^. Although few studies have investigated the physiological effects of seawater on alligators, there is emerging evidence that seawater has significant impacts on alligator endocrine physiology^2,3,5,22^. For instance, juvenile alligators ceased feeding, experienced weight loss and showed elevated plasma corticosterone and electrolytes (Na^+^, Cl^−^) when exposed to salinities between 10 and 20‰ for 4 weeks^3^ and 5 weeks^22^. Further, mortality was observed in juvenile alligators exposed to 15‰ and 20‰ seawater after 3 weeks^3^ while 4 mortalities occurred out of 10 animals exposed to 16‰ for 5 weeks^22^. In addition, exposure of juvenile alligators to 12‰ seawater for 5 weeks significantly elevated circulating levels of electrolytes (Na^+^, Cl^−^), lowered angiotensin II [a renin–angiotensin–aldosterone system (RAAS) hormone], and elevated important steroid hormones involved in stress response (glucocorticoids) and reproduction and growth (progestogens, androgens, estrogens)^2^. Long-term exposure to brackish seawater levels (12‰) therefore caused important alterations in reproductive ability while depressing the endocrine system responsible for water/salt regulation. Short-term (1 week) exposure to 12‰ seawater similarly elevated Na^+^ and Cl^−^ and glucocorticoid levels in juvenile alligators while estrogen levels decreased and androgen levels were largely unaffected^5^. Collectively, these studies demonstrated that exposure to high salinity can cause time-dependent changes in sex steroids important for growth and reproduction.

Steroid hormones are vital in controlling various physiological processes such as growth, development, reproduction, and response to stress^23–28^. Thus, steroids are commonly used as biomarkers to assess the physiological impacts of environmental or anthropogenic stressors^29^. It is well-documented that wild alligators are susceptible to endocrine disruption via exposure to contaminants such as the estrogenic pesticides dicofol, DDT, and various metabolites such as p,p’-DDE^29–31^. For instance, male juvenile alligators in the contaminated Lake Apopka had significantly greater 17β-estradiol and lower testosterone than in an uncontaminated lake, while juvenile females in the contaminated lake showed significantly elevated 17β-estradiol^29–31^. While we have significant knowledge on anthropogenic compounds, little information exists as to the effects of a natural stressor (e.g., salinization) on alligator endocrine physiology. This information is especially pertinent as changes in hydrology and saline environments have been demonstrated to negatively impact alligator populations by reducing body condition, growth, and reproductive success^32–34^. These findings further emphasize the need to elucidate how environmental factors such as salinity affects the physiology of alligators in the wild. Understanding physiological effects is crucial to the successful conservation and management of wild alligators in a changing climate.

Although we have some information on how salinity affects alligator physiology from laboratory studies, there is currently limited information^11^ regarding the effect of salinity on the physiology of juvenile alligators found in the wild. Thus, the objective of this study was to determine whether wild juvenile alligator endocrine physiology is affected by exposure to varying salinity levels. Our working hypothesis was that endocrine effects observed in laboratory-kept juvenile alligators exposed to 12‰ salinity (changes in blood biochemistry, stress hormones, sex steroids) would be reflected in wild juvenile alligators found in hyperosmotic environments. While salinity of habitats may vary spatially and temporally depending on inflow of freshwater, juvenile alligator physiology quickly changes when exposed to elevated salinity. For instance, exposure to ≥ 10‰ seawater for a 1-week period significantly increased plasma ions (Na^+^, Cl^−^) and corticosterone in juvenile alligators^3,5^, and changes in sex hormones and gonad morphology have also been observed in juvenile alligators exposed to 12‰ seawater for as little as 1 week^5^. In addition, 12‰ seawater caused behavioral changes including cessation of feeding in juvenile alligators within days of exposure (Faulkner et al., 2021, in review). Thus, we anticipated that while salinity varies throughout the environment, even short-term exposure to environmental salinity would correlate with physiological changes in wild-caught juvenile alligators.

Wild juvenile alligators were opportunistically caught and blood sampled at Rockefeller Wildlife Refuge in Grand Chenier, LA each month over the course of one year (July 2018-July 2019). Environmental salinity levels were measured at the site of capture. Hormones involved in the RAAS (angiotensin II, aldosterone), stress response (glucocorticoids: 11-deoxycortisol, corticosterone), and reproduction and growth [progestogens (pregnenolone, 17α-hydroxypregnenolone, progesterone, 17α-hydroxyprogesterone, 17α,20β-dihydroxypregnenone (DHP)), androgens (androstenedione, 5α-dihydrotestosterone, testosterone), and estrogens (estrone, 17β-estradiol, estriol)] were determined. Partial redundancy analysis was performed to determine correlation between salinity and hormones as well as various blood plasma biochemistry parameters (e.g., Na^+^, Cl^−^). This study presents new knowledge by monitoring monthly changes in a wide range of hormones and blood biochemistry parameters in male and female juvenile American alligators.

## Methods

### Wild alligator sampling and blood plasma collection

**Figure 1:**
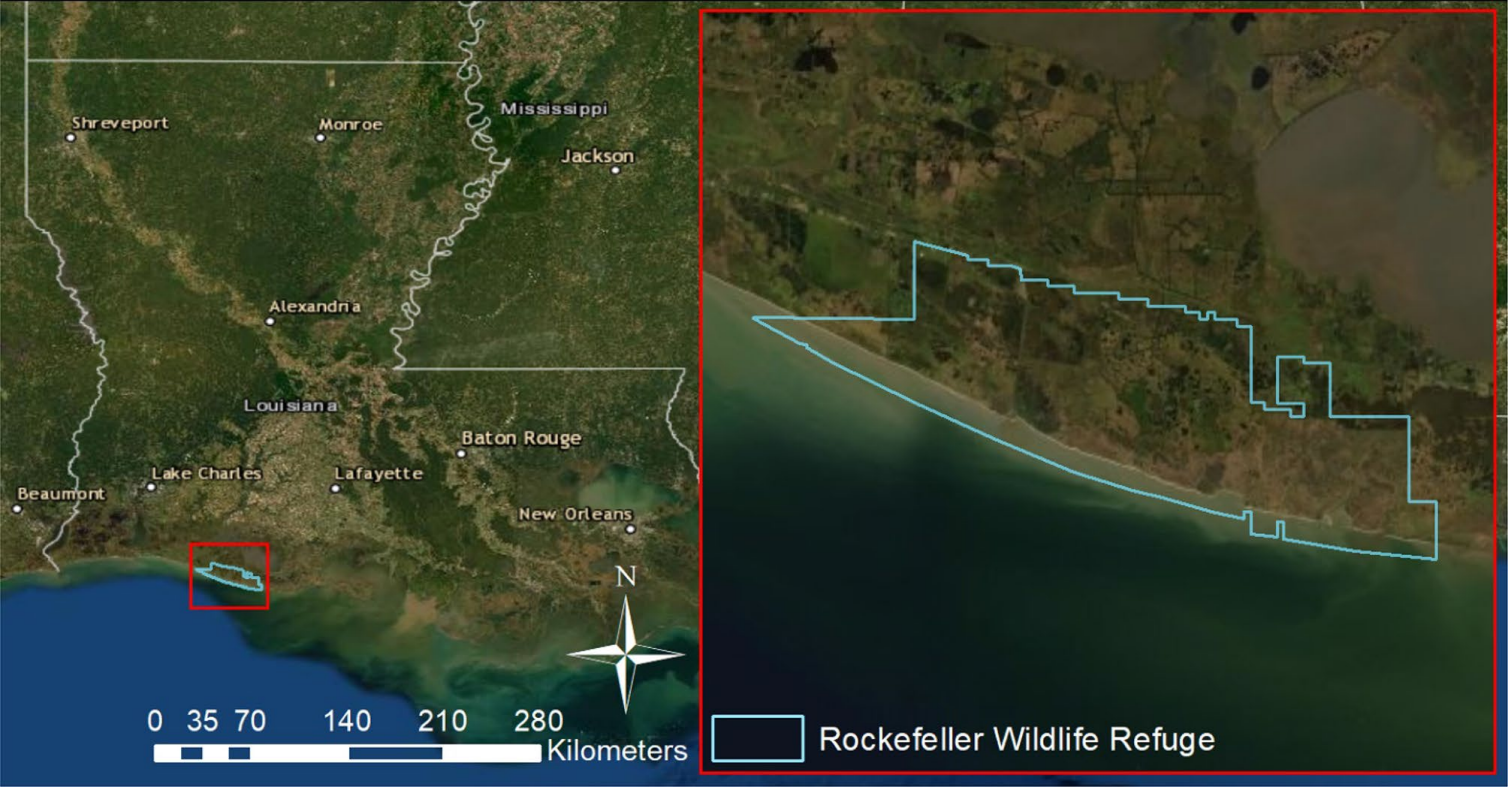
Rockefeller Wildlife Refuge in Grand Chenier, Louisiana (U.S.A.) where wild American alligators (*Alligator mississippiensis*) were sampled from various locations. Map generated using ArcMap version 10.8 (https://desktop.arcgis.com/en/arcmap/) with satellite imagery obtained from the GIS Community Maps Program. (https://www.arcgis.com/home/item.html?id=10df2279f9684e4a9f6a7f08febac2a9).

Fifty-four blood samples were opportunistically collected from juvenile alligators (< 1.8 m total length^35^) by the Louisiana Department of Wildlife and Fisheries during the day between July 2018 and July 2019 at Rockefeller Wildlife Refuge (Grand Chenier, LA) (Fig. 1). Alligators were sampled near the Rockefeller Wildlife Refuge headquarters by slowly driving gravel roads on the refuge to observe alligators, and attempting to capture alligators seen in roadside ponds, ditches, and canals. Alligators were not sampled in November, December, and January when cool temperatures during winter months limit their activity. Varying numbers of alligators were sampled per month and per sex due to the opportunistic sampling method. After alligators were located, animals were restrained and blood was quickly drawn from the post-occipital spinal venous sinus and placed in 10 ml lithium heparin vacutainers, centrifuged at 1318 × *g* for 10 min, then transferred to microfuge tubes for storage at − 20 °C until chemical analysis. Morphometrics [total length (66–178 cm), and sex (male N = 33, female N = 21)] were recorded from each animal, and salinity levels (0.4–22.2‰) of each animal’s capture site were measured using a YSI 30 salinity meter. The present study was carried out in compliance with the Animal Research: Reporting of In Vivo Experiments (ARRIVE) guidelines. All experimental protocols were carried out in accordance with the guidelines issued by the Texas A&M University’s Animal Care Committee, and all methods were approved by the Texas A&M University’s Animal Care Committee under IACUC AUP 2019-0095.

### Blood biochemistry analysis

Blood plasma biochemistry levels were measured by Texas A&M University’s Veterinary Medical Diagnostic Laboratory (College Station, TX, USA). A Beckman Coulter AU480 analyzer was used to measure Na^+^, K^+^, Cl^−^, Ca^2+^, phosphorus, uric acid, total protein, albumin, globulin, bilirubin, glucose, cholesterol, creatinine, creatine kinase, alkaline phosphatase (ALP), aspartate aminotransferase (AST), and alanine aminotransferase (ALT).

### Hormone extraction from blood plasma

Hormone extractions were performed as previously described^2,5^, with the following modifications. Plasma samples were thawed on ice, and 500 μl aliquots of plasma were spiked with surrogate standards d9-progesterone and d3-17β-estradiol. d9-Progesterone was used as the surrogate standard to quantify progesterone, 17α-hydroxyprogesterone, pregnenolone, 17α-hydroxypregnenolone, DHP, angiotensin II, aldosterone, 11-deoxycortisol, corticosterone, androstenedione, 5α-dihydrotestosterone, and testosterone, while d3-17β-estradiol was used as the surrogate standard to quantify estrone, 17β-estradiol, and estriol. The plasma was suspended in 5 ml Milli-Q water and liquid:liquid extracted twice using methyl tert-butyl ether (MTBE). Liquid:liquid extraction involved vortexing to mix the plasma with 5 ml MTBE for 10 s to allow for partitioning of analytes from the aqueous phase (plasma) to the solvent phase (MTBE). The mixture was subsequently centrifuged for 5 min at 2000 × *g* to separate the plasma and MTBE into distinct layers. Pooled MTBE layers were gently dried under nitrogen gas, and the residue was reconstituted in 50 μl of 30:70 methanol:Milli-Q water. Samples were transferred to small-volume inserts in 2 ml amber glass vials for analysis via liquid chromatography and tandem mass spectrometry (LC–MS/MS).

Prior to estrogen analysis, a 25 μl sub-aliquot of MTBE-extracted steroids was derivatized with dansyl chloride^36^. The sub-aliquot was dried under nitrogen and reconstituted in 50 μl of 1 mg ml^−1^ dansyl chloride in acetone and 50 μl of 100 mM sodium bicarbonate, and incubated at 60 °C for 3 min. The resulting dansylated estrogens (Dns-estrogens) were suspended in 500 μl of Milli-Q water and liquid:liquid extracted twice using 500 μl 1:1 hexane:ethyl acetate. Pooled solvent layers were dried under nitrogen, and residue was reconstituted in 30:70 methanol:Milli-Q water and transferred into small-volume inserts for LC–MS/MS analysis.

### LC–MS/MS analysis of steroid hormones

Chemical analyses were as described previously^2,5^, with the following modifications. The LC–MS/MS system comprised an Agilent 1260 UHPLC system and a triple-quad 6420 mass detector. Chromatography separation of steroid hormones was achieved with an Agilent Poroshell EC-C18 column (3.0 × 50 mm, 5 μm particle size). The liquid mobile phase consisted of Milli-Q water (A) and methanol (B), each containing 5 mM ammonium formate. The mobile phase gradient transitioned from 30% (B) linearly to 70% B over 3 min and from 70 to 95% in 6 min. The gradient subsequently decreased from 95 to 70% over 3 min and from 70 to 30% (initial condition) in 3 min with the flow rate maintained at 0.4 ml min^−1^ (total run-time of 15 min).

Plasma hormones were ionized in positive ion electrospray ionization (ESI +) mode using nitrogen as the desolvation gas heated to 350 °C (gas flow of 12 L min^−1^) and a capillary voltage of 3.5 kV. Hormones were detected in multiple reaction monitoring (MRM) mode with argon as the collision gas. The precursor → product ions monitored included: *m/z* (mass-to-charge ratio) 523.8 → 70.3 (angiotensin II), *m/z* 361.2 → 343.2 (aldosterone), *m/z* 347.2 → 97.2 (11-deoxycortisol), *m/z* 347.2 → 121.1 (corticosterone), *m/z* 317.3 → 299.2 (pregnenolone), *m/z* 333.2 → 315.2 (17α-hydroxypregnenolone), *m/z* 315.2 → 97.1 (progesterone), *m/z* 331.2 → 97.1 (17α-hydroxyprogesterone), m/z 333.2 → 97.1 (DHP), *m/z* 287.1 → 97.0 (androstenedione), *m/z* 291.2 → 255.1 (5α-dihydrotestosterone), *m/z* 289.2 → 97.0 (testosterone), *m/z* 324.3 → 100.2 (d9-progesterone); and dansyl chloride (Dns) derived estrogens: *m/z* 504.2 → 171.1 (Dns-estrone), *m/z* 506.2 → 171.1 (Dns-estradiol), *m/z* 522.2 → 171.1 (Dns-estriol), and *m/z* 509.3 → 171.1 (Dns-d3-estradiol).

### Statistical analyses

Normality testing was performed using Shapiro–Wilk’s test (p ≤ 0.05) and data were log_10_ transformed which ensured homogeneity prior to correlation analysis. Partial redundancy analysis (pRDA) was performed to determine correlations between physiological parameters and environmental salinity using the vegan package in R. A permutation test using 999 permutations was used to test the significance of relationships between physiological parameters and environmental salinity. Spearman’s rank-order correlation analysis was used to further test the correlation between environmental salinity and all blood biochemistry parameters and hormones. One-way ANOVAs were used to test for variation in individual hormone levels and blood biochemistry parameters in each sex by month. ANOVAs were followed by post-hoc Scheffe’s tests, which was selected as a conservative test to reduce the chances of type I error while performing a large number of analyses. Differences in physiological parameters between male and female alligators within each month were determined by two-tailed Student’s *t*-tests for parametric data and Wilcoxon tests for nonparametric data. If Wilcoxon test assumptions were not met, permutation tests using 1000 permutations were performed instead. All statistical analyses were performed using R version 3.6.3. All data shown in figures, tables, and text are mean ± SEM. Statistical significance was assumed at p ≤ 0.05.

## Results

### Alligator size and environmental salinity levels

**Figure 2:**
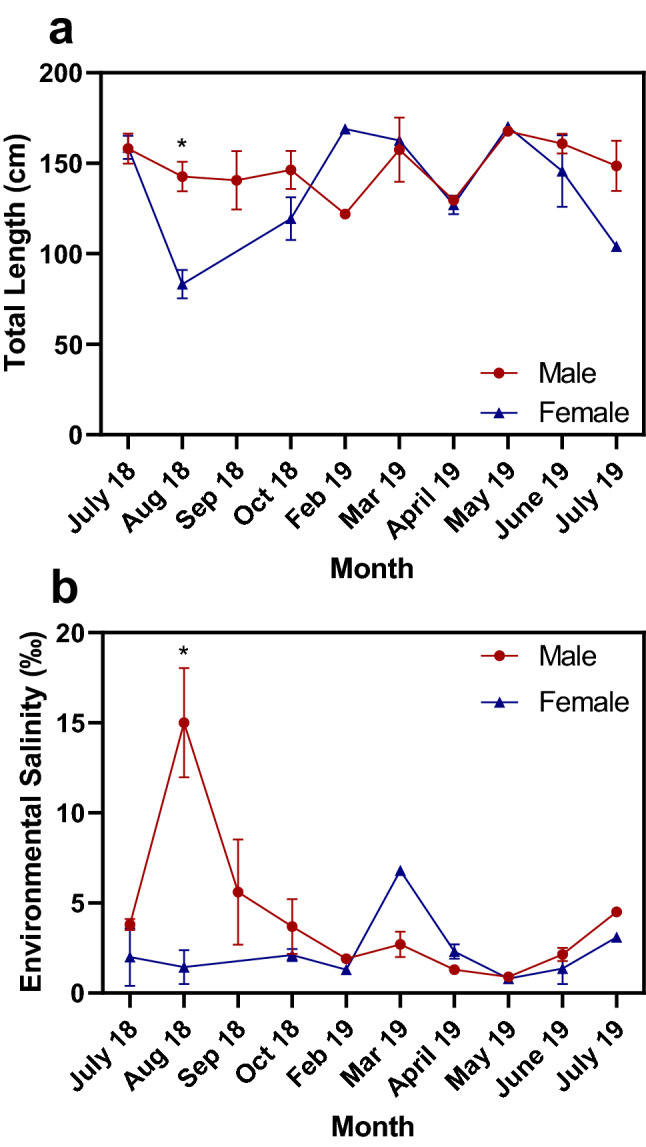
Monthly variation in total length (**a**) of wild juvenile American alligators (*Alligator mississippiensis*) and environmental salinity in which animals were located (**b**). *Denotes significant differences between males and females during the indicated month.

Total length of wild male and female juvenile alligators did not vary significantly by month of sampling with one exception. Average total length of males was significantly higher than females during August 2018 (*t*-test, p < 0.001) (Fig. 2). Similarly, no significant variance in environmental salinity at capture sites of males and female was detected between months. However, some males were found in significantly higher environmental salinity than females during August 2018 (permutation test, p = 0.022), corresponding with the (not significant) trend of the highest average environmental salinity occurring in August 2018 (Fig. 2). In total, 7 of the 33 juvenile males were found in hyperosmotic salinities of 10‰ or greater^3,22^ during the present study. These comprised 5 males found in water between 16.2 and 22.2‰ during August 2018, one male in 10.9‰ in September 2018, and one male in 12.4‰ during October 2018. On the other hand, none of the 21 juvenile females were found in hyperosmotic environments.

### Multi-variate analysis of blood chemistry and hormones

**Figure 3:**
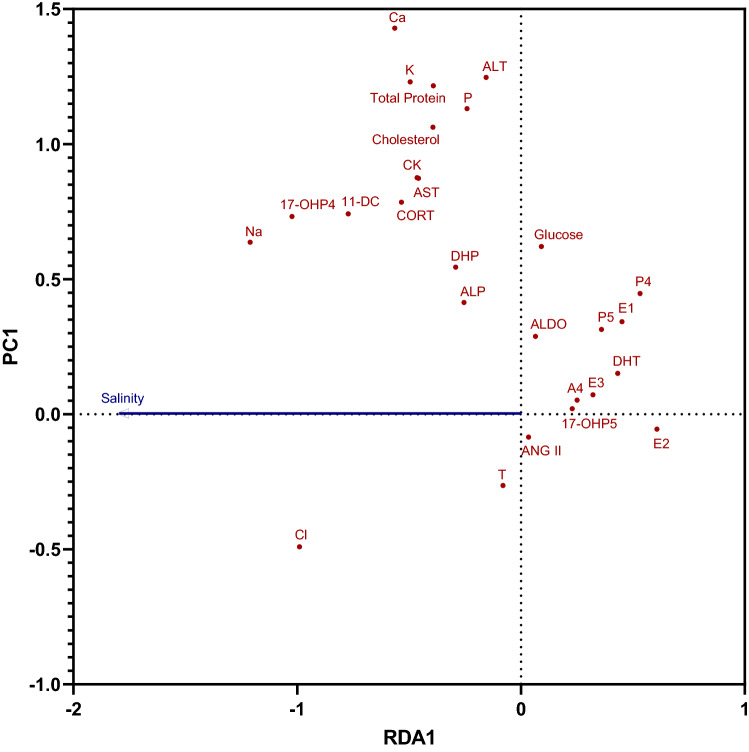
Partial redundancy analysis using environmental salinity in which American alligators (*Alligator mississippiensis*) were found as a predictor and holding total length, sex, and month constant. Abbreviated are angiotensin II (ANG II), aldosterone (ALDO), 11-deoxycortisol (11-DC), corticosterone (CORT), pregnenolone (P5), 17α-hydroxypregnenolone (17-OHP5), progesterone (P4), 17α-hydroxyprogesterone (17-OHP4), 17α,20β-dihydroxypregnenone (DHP), androstenedione (A4), 5α-dihydrotestosterone (DHT), testosterone (T), estrone (E1), 17β-estradiol (E2), estriol (E3), sodium (Na), chloride (Cl), potassium (K), calcium (Ca), phosphorus (P), alkaline phosphatase (ALP), aspartate aminotransferase (AST), and alanine aminotransferase (ALT).

The pRDA found significant correlation between environmental salinity and blood chemistry parameters (p = 0.003), but a low adjusted r^2^ of 0.031 indicates a low proportion of variance in blood chemistry levels overall is explained by environmental salinity. Blood plasma Na^+^ and Cl^−^ appear positively correlated with environmental salinity, albeit not very strongly, while 17α-hydroxyprogesterone, 11-deoxycortisol, and corticosterone are more weakly, but positively correlated with salinity (Fig. 3). On the other hand, 17α-hydroxypregnenolone, androstenedione, 5α-dihydrotestosterone, 17β-estradiol, and estriol appear negatively, but weakly correlated with environmental salinity. Other parameters are closer to 90-degree angles compared with salinity with respect to the origin, signifying weaker correlation.

### Correlations of blood biochemistry and hormones with environmental salinity levels

**Figure 4:**
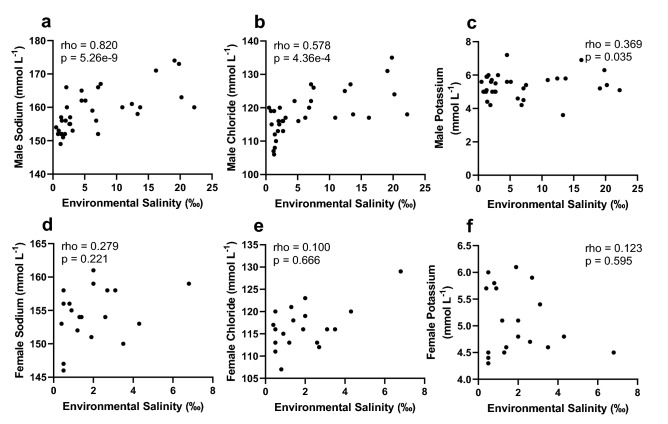
Spearman rank correlation between environmental salinity and plasma sodium (**a**,**d**), chloride (**b**,**e**), and potassium (**c**,**f**) in juvenile male and female American alligators (*Alligator mississippiensis*). *rho* Spearman’s rho, *p* significance of correlation.

**Figure 5:**
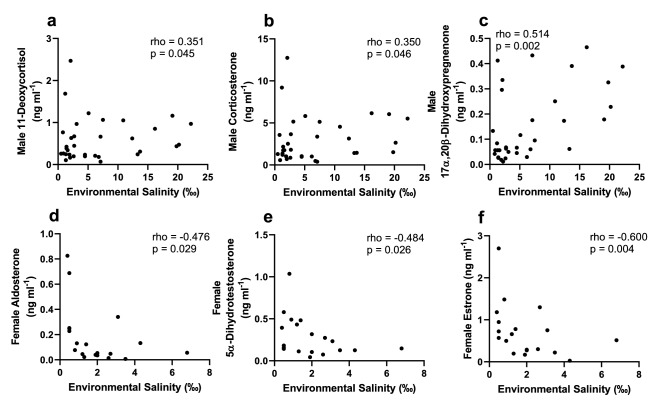
Spearman rank correlation between environmental salinity and plasma hormone levels found to be significant (p < 0.05) in juvenile male (**a**–**c**) and juvenile female (**d**–**f**) American alligators (*Alligator mississippiensis*). *rho* Spearman’s rho, *p* significance of correlation.

Spearman rank correlations showed significant, positive correlations between environmental salinity and both plasma Na^+^ (p < 0.001) and Cl^−^ (p < 0.001) in juvenile male alligators, while plasma K^+^ was also positively, albeit weakly (p = 0.035) correlated with environmental salinity (Fig. 4). In addition, environmental salinity of males was also positively, but weakly correlated with plasma 11-deoxycortisol (p = 0.045) and corticosterone (p = 0.046), while showing a stronger positive correlation with DHP (p = 0.002) (Fig. 5). Although female alligator ion concentrations in plasma were not correlated with environmental salinity, environmental salinity was significantly negatively correlated with plasma aldosterone (p = 0.045), 5α-dihydrotestosterone (p = 0.046), and estrone (p = 0.002) levels in females (Fig. 5).

### Differences in blood biochemistry parameters between months and sex

Plasma ion concentrations (Na^+^, K^+^, Cl^−^, Ca^2+^, P) did not vary significantly by month in male or female juvenile alligators. However, significant sex differences were detected in various blood biochemistry parameters. For instance, males had significantly greater blood Na^+^ (*t*-test, p = 0.003), Ca^2+^ (*t*-test, p = 0.014), and P (*t*-test, p = 0.033) levels compared with females during August 2018 (Table 1) when salinities were higher due to lower rainfall. Among plasma biochemistry parameters, ALP was the only parameter to vary significantly by month as male ALP in October 2018 was significantly lower than in both July 2018 (p = 0.036) and June 2019 (p = 0.017) (Table 2). Male juvenile alligators had significantly greater creatine kinase levels in plasma compared with females in August 2018 (*t*-test, p = 0.040) (Table 2). During the same month of August, females had significantly lower blood plasma ALT (*t*-test, p = 0.050) and significantly greater AST:ALT ratios than in males (permutation test, p = 0.042) (Table 2). Plasma albumin, globulin, creatinine, and uric acid levels were below the detection limit in most animals.

**Table 1:** Plasma ion concentrations in juvenile American alligators (*Alligator mississippiensis*) sampled in the wild.

Ion	Sex	July 2018	Aug 2018	Sep 2018	Oct 2018	Feb 2019	Mar 2019	April 2019	May 2019	June 2019	July 2019
Sodium (mmol l^−1^)	Male	158.50 ± 0.50	166.86 ± 3.29	158.00 ± 3.06	156.63 ± 1.93	152.00	159.50 ± 3.50	157.00	153.00	154.33 ± 1.41	163.50 ± 1.50
	Female	155.50 ± 2.50	150.50 ± 2.40*	–	155.00 ± 1.71	154.00	159.00	154.50 ± 3.50	156.00	157.33 ± 0.67	153.00
Potassium (mmol l^−1^)	Male	5.50 ± 1.00	5.61 ± 0.27	5.43 ± 0.22	5.16 ± 0.17	4.20	5.85 ± 0.25	5.90	5.00	5.32 ± 0.19	6.40 ± 0.80
	Female	5.90 ± 0.20	4.80 ± 0.32	–	4.82 ± 0.09	4.50	4.50	6.00 ± 0.10	5.80	5.30 ± 0.44	6.00
Na:K ratio	Male	29.80 ± 5.30	30.03 ± 1.16	29.13 ± 0.67	30.53 ± 0.90	35.30	27.30 ± 0.60	26.60	30.60	29.22 ± 1.06	25.90 ± 3.00
	Female	26.35 ± 0.45	31.68 ± 1.50	–	32.23 ± 0.55	34.20	35.30	25.80 ± 1.00	26.90	30.10 ± 2.46	25.50
Chloride (mmol l^−1^)	Male	115 ± 5.00	123.86 ± 2.59	117.33 ± 0.88	116.38 ± 2.60	113.00	116.00 ± 0.00	106.00	115.00	115.67 ± 1.48	122.00 ± 0.00
	Female	112.50 ± 4.50	117.50 ± 1.85	–	117.00 ± 1.57	121.00	129.00	114.00 ± 2.00	107.00	115.67 ± 2.60	117.00
Calcium (mg dl^−1^)	Male	10.45 ± 0.65	11.24 ± 0.39	10.17 ± 0.34	10.51 ± 0.41	11.00	11.95 ± 0.75	12.30	9.90	11.12 ± 0.25	11.35 ± 0.55
	Female	11.10 ± 0.80	9.78 ± 0.28*	–	10.62 ± 0.37	10.00	9.20	10.65 ± 0.35	10.90	11.50 ± 0.26	10.80
Phosphorus (mg dl^−1^)	Male	5.55 ± 1.25	5.87 ± 0.65	4.83 ± 0.32	3.98 ± 0.29	3.40	6.00 ± 0.90	4.40	4.70	6.40 ± 0.39	5.40 ± 1.40
	Female	3.80 ± 0.20	3.80 ± 0.50*	–	4.18 ± 0.19	3.00	3.50	5.65 ± 0.95	5.90	6.27 ± 0.57	4.40
Sample size	Male	2	7	3	8	1	2	1	1	6	2
	Female	2	4	0	6	1	1	2	1	3	1

*Significant difference between males and females during the indicated month (p < 0.05). Values shown are averages ± SEM. SEM is not shown in cases including only one animal.

**Table 2:** Plasma biochemistry parameters in juvenile American alligators (*Alligator mississippiensis*) sampled in the wild.

Blood chemistry parameter	Sex	July 2018	Aug 2018	Sep 2018	Oct 2018	Feb 2019	Mar 2019	April 2019	May 2019	June 2019	July 2019
Total protein (mg dl^−1^)	Male	4.20 ± 1.20	4.39 ± 0.37	4.17 ± 0.32	4.26 ± 0.28	5.70	5.00 ± 0.50	5.30	4.00	4.22 ± 0.30	4.20 ± 0.60
	Female	4.95 ± 0.15	3.63 ± 0.23	–	4.68 ± 0.39	4.00	3.00	5.15 ± 0.15	5.60	4.40 ± 0.21	4.70
Bilirubin (mg dl^−1^)	Male	0.10 ± 0.00	0.10 ± 0.00	0.10 ± 0.00	0.10 ± 0.00	0.10	0.10 ± 0.00	0.10	0.10	0.10 ± 0.00	0.10 ± 0.00
	Female	0.10 ± 0.00	0.10 ± 0.00	–	0.10 ± 0.00	0.10	0.10	0.10 ± 0.00	0.10	0.10 ± 0.00	0.10
Albumin (mg dl^−1^)	Male	< 1.50†	1.54 ± 0.04	< 1.50†	1.56 ± 0.04	< 1.50†	1.65 ± 0.15	< 1.50†	< 1.50†	< 1.50†	< 1.50†
	Female	1.55 ± 0.05	1.53 ± 0.03	–	1.60 ± 0.05	< 1.50†	< 1.50†	1.55 ± 0.05	1.70	< 1.50†	< 1.50†
Globulins (mg dl^−1^)	Male	3.90(< 1.50†)	3.50 ± 0.30	< 1.50†	3.30 ± 0.12	< 1.50†	3.70(< 1.50†)	3.80	< 1.50†	4.00(< 1.50†)	< 1.50†
	Female	3.20(< 1.50†)	2.70(< 1.50†)	–	3.28 ± 0.48	< 1.50†	1.50	3.70(< 1.50†)	3.90	< 1.50†	< 1.50†
A:G ratio	Male	0.40	0.47 ± 0.03	–	0.50 ± 0.00	–	0.50	0.40	–	0.40	–
	Female	0.50	0.60	–	0.55 ± 0.06	–	–	0.40	0.40	–	–
Creatinine (mg dl^−1^)	Male	< 0.20†	0.37 ± 0.09	< 0.20†	0.23 ± 0.02	< 0.20†	< 0.20†	< 0.20†	< 0.20†	< 0.20†	0.25 ± 0.05
	Female	0.30 ± 0.00	< 0.20†	–	0.27 ± 0.04	< 0.20†	< 0.20†	< 0.20†	< 0.20†	< 0.20†	0.30
Creatine kinase (U l^−1^)	Male	792.00 ± 300.00	687.29 ± 205.27	362.67 ± 49.24	321.00 ± 44.58	381.00	786.50 ± 56.50	677.00	411.00	945.50 ± 274.56	551.00 ± 130.00
	Female	227.50 ± 52.5	149.75 ± 6.97*	–	691.17 ± 297.96	1984.00	1264.00	693.50 ± 82.50	670.00	525.00 ± 43.10	1212.00
ALP (U l^−1^)	Male	22.50 ± 0.50^a^	14.57 ± 2.29^a,b^	11.00 ± 0.58^a,b^	7.75 ± 0.70^b^	5.00^a,b^	18.50 ± 0.50^a,b^	22.00^a,b^	14.00^a,b^	17.67 ± 2.33^a^	18.00 ± 0.00^a,b^
	Female	13.00 ± 3.00	11.25 ± 1.93	–	7.83 ± 0.79	7.00	29.00	19.00 ± 2.00	19.00	17.00 ± 3.79	22.00
AST (U l^−1^)	Male	167.00 ± 45.00	185.14 ± 39.58	152.67 ± 25.83	162.00 ± 9.52	155.00	226.00 ± 52.00	188.00	156.00	187.00 ± 15.11	183.50 ± 31.50
	Female	163.50 ± 15.50	188.00 ± 7.36	–	222.33 ± 35.38	136.00	129.00	218.50 ± 7.50	215.00	178.67 ± 4.91	240.00
ALT (U l^−1^)	Male	31.00 ± 2.00	36.00 ± 6.40	35.33 ± 5.04	40.75 ± 4.51	29.00	42.50 ± 6.50	60.00	35.00	54.33 ± 6.86	42.50 ± 8.50
	Female	39.50 ± 0.50	18.75 ± 4.17*	–	45.67 ± 5.10	38.00	22.00	48.00 ± 17.00	56.00	43.00 ± 3.06	74.00
AST:ALT ratio	Male	5.32 ± 1.11	5.97 ± 1.37	4.31 ± 0.31	4.20 ± 0.35	5.34	5.25 ± 0.42	3.13	4.46	3.55 ± 0.27	4.35 ± 0.13
	Female	4.15 ± 0.45	11.31 ± 1.92*	–	4.98 ± 0.66	3.58	5.86	5.15 ± 1.67	3.84	4.18 ± 0.18	3.24
Glucose (mg dl^−1^)	Male	89.00 ± 4.00	96.43 ± 7.56	108.33 ± 2.85	73.13 ± 4.06	49.00	88.00 ± 3.00	88.00	82.00	95.67 ± 3.63	80.00 ± 13.00
	Female	71.50 ± 3.50	96.75 ± 2.39	–	77.67 ± 4.48	60.00	53.00	95.00 ± 12.00	93.00	106.67 ± 12.71	78.00
Cholesterol (mg dl^−1^)	Male	55.50 ± 25.50	75.14 ± 10.57	75.33 ± 7.86	61.75 ± 7.14	65.00	87.00 ± 5.00	78.00	37.00	73.50 ± 5.02	89.00 ± 35.00
	Female	64.00 ± 11.00	77.25 ± 14.05	–	73.50 ± 12.39	27.00	44.00	73.00 ± 30.00	61.00	93.33 ± 22.58	54.00
Uric acid (mg dl^−1^)	Male	1.70 ± 0.20	2.46 ± 0.55	1.70 ± 0.20	1.65 ± 0.11	1.70	< 1.50†	1.80	< 1.50†	< 1.50†	< 1.50†
	Female	1.60 ± 0.10	< 1.50†	–	1.60 ± 0.10	< 1.50†	< 1.50†	1.55 ± 0.05	< 1.50†	< 1.50†	< 1.50†
Sample size	Male	2	7	3	8	1	2	1	1	6	2
	Female	2	4	0	6	1	1	2	1	3	1

Dissimilar letters show significant differences between months within the given sex. *Significant difference between males and females during the indicated month (p < 0.05). †Values below detection limit. Values shown are averages ± SEM. SEM is not shown in cases including only one animal. Outliers excluded from analyses are shown in parentheses.

### Differences in hormone levels between months and sex

Plasma hormone levels varied significantly during certain months, such as corticosterone levels in male alligators which were significantly greater in July 2018 than in March 2019 (p = 0.042) which can be explained by higher salinities in summer compared to winter. Male pregnenolone levels in September 2018 were also significantly higher than in June 2019 (p = 0.039), and 17α-hydroxypregnenolone in females was greater in August 2018 compared with females in October 2018 (p = 0.032). Male progesterone levels were also significantly lower in October 2018 than in both July 2018 (p = 0.048) and June 2019 (p = 0.021). Furthermore, testosterone levels in juvenile male alligators were significantly greater in March 2019 compared with July 2018 (p = 0.016) and August 2018 (p = 0.050). Differences in plasma concentrations of two hormones were also detected between sexes, including angiotensin II which was significantly greater in juvenile male alligators than in females during October 2018 (Wilcoxon test, p = 0.038), while in August 2018 females had greater plasma 17α,20β-dihydroxypregnenone than males (*t*-test, p < 0.001) (Table 3).

**Table 3:** Plasma hormone levels in wild juvenile American alligators (*Alligator mississippiensis*).

Hormone	Sex	July 2018	Aug 2018	Sep 2018	Oct 2018	Feb 2019	Mar 2019	April 2019	May 2019	June 2019	July 2019
Angiotensin II (ng ml^−1^)	Male	1.15	0.26 ± 0.03	1.21 ± 0.65	0.45 ± 0.29	0.38	0.43 ± 0.14	0.06	1.05	0.19 ± 0.07	0.05 ± 0.01
	Female	1.12 ± 0.19	0.66 ± 0.53	–	0.21 ± 0.16*	0.16	0.16	0.13 ± 0.02	2.26	0.11 ± 0.04	0.02
Aldosterone (ng ml^−1^)	Male	0.16 ± 0.07	0.28 ± 0.08	0.17 ± 0.06	0.11 ± 0.04	0.10	0.12 ± 0.08	0.02	0.39	0.25 ± 0.07	0.18 ± 0.09
	Female	0.46 ± 0.37	0.35 ± 0.12	–	0.05 ± 0.02	0.02	0.06	0.04 ± 0.00	0.08	0.27 ± 0.03	0.10
11-Deoxycortisol (ng ml^−1^)	Male	1.91 ± 0.40	0.95 ± 0.32	1.01 ± 0.13	0.70 ± 0.16	0.24	0.23 ± 0.07	0.25	0.26	0.48 ± 0.08	0.22 ± 0.02
	Female	0.56 ± 0.16	0.40 ± 0.14	–	1.08 ± 0.20	0.16	0.33	0.3 ± 0.18	0.33	1.1 ± 0.23	0.97
Corticosterone (ng ml^−1^)	Male	10.40 ± 1.71^a^	5.34 ± 1.66^a,b^	4.64 ± 0.65^a,b^	3.49 ± 0.92^a,b^	1.02^a,b^	0.56 ± 0.14^b^	1.21^a,b^	0.56^a,b^	2.42 ± 0.48^a,b^	1.01 ± 0.04^a,b^
	Female	3.29 ± 1.02	1.64 ± 0.54	–	5.15 ± 1.04	0.12	0.17	1.59 ± 1.03	1.17	4.99 ± 1.14	5.15
Pregnenolone (ng ml^−1^)	Male	17.97 ± 3.62^a,b^	28.56 ± 9.79^a,b^	42.42 ± 3.17^a^	20.78 ± 4.86^a,b^	9.92_a,b_	5.18 ± 0.30^a,b^	3.13^a,b^	2.73^a,b^	4.77 ± 1.21^b^	7.33 ± 1.45^a,b^
	Female	15.6 ± 4.88	21.78 ± 6.41	–	37.28 ± 16.33	8.25	3.83	3.18 ± 0.88	26.61	7.37 ± 3.20	1.35
17α-Hydroxypregnenolone (ng ml^−1^)	Male	425.03 ± 67.30	342.28 ± 48.74	318.87 ± 119.43	141.87 ± 17.25	122.02	147.44 ± 15.13	157.41	382.25	217.25 ± 33.41	251.08 ± 36.72
	Female	255.93 ± 68.64^a,b^	466.18 ± 71.93^a^	–	107.72 ± 26.21^b^	118.06^a,b^	152.95^a,b^	94.93 ± 7.09^a,b^	321.09^a,b^	274.63 ± 87.88^a,b^	183.53^a,b^
Progesterone (ng ml^−1^)	Male	0.79 ± 0.03^a^	0.35 ± 0.08^a,b^	0.28 ± 0.02^a,b^	0.17 ± 0.02^b^	0.10^a,b^	0.30 ± 0.08^a,b^	0.09^a,b^	1.21^a,b^	0.58 ± 0.09^a^	0.17 ± 0.05^a,b^
	Female	0.24 ± 0.04	0.18 ± 0.08	–	0.20 ± 0.10	0.15	0.18	0.10 ± 0.05	0.82	0.76 ± 0.22	1.22
17α-Hydroxyprogesterone (ng ml^−1^)	Male	0.41	0.62 ± 0.27	1.06 ± 0.29	0.60 ± 0.30	0.10	0.08 ± 0.04	0.12	0.11	0.30 ± 0.07	0.15 ± 0.02
	Female	0.63 ± 0.34	0.28 ± 0.09	–	0.76 ± 0.15	0.03	0.07	0.13 ± 0.10	0.28	0.46 ± 0.19	0.74
17α,20β-Dihydroxypregnenone(ng ml^−1^)	Male	0.30 ± 0.02	0.36 ± 0.04	0.14 ± 0.06	0.11 ± 0.05	0.02	0.18 ± 0.12	0.06	0.06	0.21 ± 0.13	0.06 ± 0.01
	Female	0.18 ± 0.05	0.08 ± 0.01*	–	0.06 ± 0.01	0.09	0.04	0.02 ± 0.01	0.05	0.12 ± 0.02	0.05
Androstenedione (ng ml^−1^)	Male	0.10 ± 0.06	0.15 ± 0.09	0.25 ± 0.23	0.11 ± 0.07	0.02	1.13 ± 0.52	0.04	0.13	1.14 ± 0.49	0.24 ± 0.16
	Female	0.07 ± 0.02	0.03 ± 0.02	–	0.05 ± 0.02	0.02	0.03	0.05 ± 0.04	0.46	0.20 ± 0.04	0.03
5α-Dihydrotestosterone (ng ml^−1^)	Male	0.43 ± 0.30	0.70 ± 0.37	0.46 ± 0.18	0.55 ± 0.20	0.06	2.52 ± 0.69	0.10	0.21	0.72 ± 0.17	0.25 ± 0.04
	Female	0.26 ± 0.14	0.32 ± 0.11	–	0.26 ± 0.07	0.11	0.15	0.16 ± 0.12	1.04	0.19 ± 0.02*	0.05
Testosterone (ng ml^−1^)	Male	1.00 ± 0.09	5.70 ± 1.62	10.55 ± 7.40	8.06 ± 2.70	1.56	–	0.86	7.30	25.74 ± 5.98	8.39 ± 4.29
	Female	9.15 ± 4.24	16.72 ± 9.12	–	10.12 ± 2.05	3.34	6.64	4.10 ± 2.44	28.38	8.63 ± 0.75*	2.03
Estrone (ng ml^−1^)	Male	0.32 ± 0.14	1.31 ± 0.32	0.24 ± 0.07	0.65 ± 0.23	0.45	0.56 ± 0.09	1.72	0.99	0.72 ± 0.06	0.51 ± 0.04
	Female	0.59 ± 0.07	0.41 ± 0.08	–	1.10 ± 0.28	0.45	0.46	1.19 ± 0.53	0.99	0.82 ± 0.07	0.65
17β-Estradiol (ng ml^−1^)	Male	1.65 ± 0.10	1.48 ± 0.04	2.31 ± 0.08	1.41 ± 0.35	1.68	1.51 ± 0.11	0.38	1.54	1.11 ± 0.10	0.71 ± 0.09
	Female	1.64 ± 0.04	1.73 ± 0.31	–	0.42 ± 0.30	1.93	2.01	1.00 ± 0.81	2.42	1.06 ± 0.15	0.57
Estriol (ng ml^−1^)	Male	0.14 ± 0.03	0.82 ± 0.21	0.81 ± 0.18	2.28 ± 0.64	0.24	0.18 ± 0.00	0.55	0.33	1.12 ± 0.36	1.85 ± 0.84
	Female	0.36	1.44 ± 0.40	–	1.57 ± 0.39	0.06	0.07	0.19 ± 0.08	0.20	1.57 ± 0.44	1.99
Sample size	Male	2	7	3	8	1	2	1	1	6	2
	Female	2	4	0	6	1	1	2	1	3	1

Dissimilar letters show significant differences between months for the given sex. *Significant difference between males and females during the indicated month (p < 0.05). †Values below detection limit. Values shown are averages ± SEM. SEM is not shown in cases where data was only obtained from one animal.

## Discussion

During this study, opportunistically sampled animals were most commonly found in environmental salinities considered hypoosmotic to alligator bodily fluids. This assumption is based on previous studies which have estimated an environmental salinity level of 8‰ to be hypoosmotic to alligator body fluids with environmental salinities of 10‰ or greater being considered hyperosmotic^3,22^. Correlating the opportunistic sampling of male and female alligators throughout the year of 2018–2019 (July–July), no clear trend between salinity levels at capture and sex was apparent. It is noteworthy, however, that more juvenile males were captured in hyperosmotic environments in August 2018 while female alligators were not sampled from any hyperosmotic areas in this study. The reason why we captured smaller males in these hyperosmotic areas may be due to territoriality of alligators. For instance, large male alligators are very territorial and will defend their territory against other males^37,38^. This behavior often results in smaller and sexually immature males being pushed out of favorable areas^37^ by the territory holder and into less favorable areas (e.g., higher salinity environments). However, there is evidence that juvenile alligator populations in coastal Louisiana are significantly male-biased with 58% males of 3,000 juveniles collected over 6 years^39^, which can further explain the greater number of males in the present study. While sex differences in salinity tolerance could be a contributing factor, there were no significant differences in plasma biochemistry and hormone levels between males and females exposed to 12‰ seawater for 1 week^5^ and it is therefore more likely that the observed trend was due to behavioral than physiological effects.

A review of climate data (https://www.ncdc.noaa.gov/cdo-web/) from Rockefeller Wildlife Refuge during the sampling period of July 2018–July 2019 showed that there was lower rainfall during August 2018 (13.1 cm) than average during the sampling period (15.9 cm). In addition, August 2018 had high average temperature (28.4 °C) compared with other months during the sampling period (21.5 °C). Drought conditions brought on by low rainfall can be exacerbated by high evaporation rates due to high temperature, which correlates well with the highest salinity levels recorded during that month compared with other months in 2018–2019. It is possible that during dry conditions as seen in August 2018, fewer freshwater habitats may be available, or are occupied by large males as discussed above. Therefore, it is likely that young male alligators utilize less favorable, high-salinity environments in the process of avoiding larger males.

Alligator habitat use is highly complex and is driven by a number of factors including prey distribution and environmental salinity^40–42^, in addition to social interactions^37^. Individual niche specialization can also play a role, i.e., individuals within a population can show variable habitat use and foraging strategies independent of sex, size, and age^40,43,44^. While there is evidence that larger alligators favor freshwater habitats^40,45^, adult alligators are more able to endure salt stress to forage in higher-salinity areas. For example, adult alligators were observed to be more likely than juveniles to utilize high-salinity habitats in the Shark River Estuary in Florida^46^, and large alligators can be seen making offshore excursions into full strength seawater^47^. On the other hand, previous data also demonstrate that sub-adult males tend to utilize higher-salinity habitats in the Shark River Estuary compared with larger adult males which were mainly found in low salinity areas^40^. This use of high-salinity habitats by smaller alligators can be explained by the seeking of prey-rich areas at the cost of salt stress, as well as by smaller alligators evading territories of larger males^40,43^. These studies further highlight the complexity of alligator behavior and habitat use. For example, high variability in adult alligator movements driven by modified environmental salinity has been observed following Hurricane Irma^48^. Responses after the hurricane included alligators moving into river channels or travelling downstream, both likely due to changes in prey distribution, while others showed no change in habitat use. Since alligator behavior can be highly variable and complex, further studies are warranted to better understand the driving factors behind the use of saline habitats by young alligators^48^.

Average plasma Na^+^ levels in juvenile wild alligators (157.26 ± 0.86 mmol l^−1^) corresponded well with values obtained in laboratory-kept juvenile alligators [(148.3 ± 0.81 mmol l^−1^)^2^, (150.9 ± 0.58 mmol l^−1^)^5^], and wild juvenile (140.3 ± 0.69 mmol l^−1^)^49^ and adult alligators (146 mmol l^−1^)^50^. In addition, plasma Cl^-^ (117.22 ± 0.80 mmol l^−1^) and K^+^ (5.33 ± 0.09 mmol l^−1^) levels were similarly comparable to previously published values from juvenile laboratory-kept alligators and wild juvenile and adult alligators [Cl^-^ (117.00 ± 1.14 mmol l^−1^)^2^, (114.0 ± 2.20 mmol l^−1^)^5^, (110 mmol l^−1^)^50^, K^+^ (5.24 ± 0.17 mmol l^−1^)^2^, (5.06 ± 0.13 mmol l^−1^)^5^, (4.9 ± 0.11 mmol l^−1^)^49^, (3.8 mmol l^−1^)^50^].

Wild juvenile male alligators sampled from a range of environmental salinities showed a significant positive correlation between salinity and plasma Na^+^ and Cl^−^ levels. Like most animals, alligators are unable to prevent Na^+^ influx when in saline environments. For example, unfed alligators ranging in size from 310–586 g showed an average Na^+^ influx of ~ 10.8 μmol 100 g^−1^ h^−1^ when exposed to 35‰ for up to 4 h (while water efflux was 0.25 ml 100 g^−1^ h^−1^)^51^. Freshwater also has significant effects on electrolyte balance, as net Na^+^ and K^+^ loss occurs in solutions up to 1 mmol L^−1^ Na^+^ and 0.4 mmol L^−1^ K^+^ in freshwater^52,53^. Additionally, hatchlings (0.03–0.07 g) exhibit a whole-body Na^+^ efflux of 3.9 μmol 100 g^−1^ h^−1^ in freshwater^52^. Thus, as alligator integument is not impermeable to fluxes of electrolytes, the positive and significant correlations in wild alligators are therefore likely due to passive influx of ions through the integument and mucous membranes, while water loss also contributed to elevated ion levels as seen in laboratory-kept alligators^5^.

There were no significant trends in any other blood biochemistry parameters (Tables 1, 2, Fig. 3) dependent on environmental salinity. However, while uric acid was not detected in most samples, uric acid was more consistently above the detection limit and tended to be higher in August 2018 males (not significant), which were found in higher saline environments. Although this trend is not significant, it does correlate well with findings from laboratory studies where uric acid significantly increased in juvenile alligators exposed for 1 and 5 weeks to 12‰ seawater^2,5^.

While plasma K^+^ in wild juvenile males significantly and positively correlated with environmental salinity, the low variability in male plasma K^+^ across salinity levels would suggest activation of aldosterone to promote K^+^ excretion. However, aldosterone in males was not correlated with salinity, which corresponds with a lack of increased aldosterone in laboratory-kept juvenile alligators exposed to 12‰ seawater^2,5^. As suggested previously^2,5^, the mechanisms controlling K^+^ excretion and the role of aldosterone in osmoregulation in seawater -exposed alligators are not fully understood and require further investigation. This observation is further supported by data from female alligators which albeit were not found in hyperosmotic salinity, but data analysis revealed aldosterone to be strongly and negatively correlated with environmental salinity in females. The observation that aldosterone is higher in hypoosmotic environments further demonstrates aldosterone does not regulate water reabsorption in alligators and supports the previous argument for a yet unknown but non-osmoregulatory role of aldosterone in alligators. Similar to aldosterone, the other RAAS hormone angiotensin II was not affected by hyperosmotic salinity levels in the present study. This is in contrast to laboratory studies on 1- and 5-week seawater-exposed juvenile alligators where angiotensin II was significantly decreased after 5 weeks in 12‰ and reduced after 1 week in 12‰^2,5^. Therefore, the present study provides further evidence that RAAS was depressed in juvenile alligators exposed to hyperosmotic salinities.

We detected a significant, weakly positive correlation between environmental salinity and the glucocorticoids corticosterone and 11-deoxycortisol in wild juvenile male alligators. However, glucocorticoid levels of animals found in hyperosmotic water (< 6 ng ml^−1^ corticosterone, < 2 ng ml^−1^ 11-deoxycortisol) did not exceed those previously seen in unstressed alligators [11.91 ± 2.74 ng ml^−1^ corticosterone and 2.22 ± 0.52 ng ml^−1^ 11-deoxycortisol^5^, 5.5 ± 0.8 ng ml^−1^ corticosterone^3^]. Thus, the present study does not show elevated corticosterone or 11-deoxycortisol resulting from salinity exposure. These findings are likely due to confounding factors in the wild, such as variable or less time spent in hyperosmotic environments. While corticosterone is an indicator of stress in reptiles^54^, corticosterone has been implicated to play an osmoregulatory role in lizard species such as sand goannas (*Varanus gouldii*)^55^ and desert iguanas (*Dipsosaurus dorsalis*)^56,57^ by promoting Na^+^ excretion in kidneys. Since salt stress has been well documented to be associated with elevated corticosterone in wild and captive alligators^2,3,5,11^, it is likely that glucocorticoids play an osmoregulatory role in crocodilians. As previously speculated, it is possible corticosterone may have mineralocorticoid effects since elevated glucocorticoid levels were not seen alongside increased plasma glucose, suggesting a nontraditional effect of corticosterone^5^.

Androgen (androstenedione, 5α-dihydrotestosterone, testosterone) and estrogen (estrone, 17β-estradiol, estriol) levels in wild alligators were not correlated with hyperosmotic environmental salinity in males. This result is likely due to less time of exposure in the wild. Previous lab studies showed time-dependent dynamic changes in sex hormones, in which 5-week seawater exposure significantly increased testosterone and estrogen levels^2^, whereas 1-week exposure significantly decreased estrone and 17β-estradiol levels^5^. Conversely, in females we found significant negative correlations between environmental salinity (0.4–6.8‰) and 5α-dihydrotestosterone and estrone. However, these significant correlations are likely an artifact of seasonal variation in sex hormones since higher values occurred during the months of April, May, July, August, and October. As these months have been associated with increased female testosterone and 17β-estradiol levels^58–60^, it is more likely these higher levels are explained by seasonal cycling than by hypoosmotic environments. The fact that sex steroid levels were not explained by differences in salinity in the present study could be due to the many confounding factors found in the wild compared with laboratory-based studies, small sample sizes, and unknown duration of exposure to environmental salinity.

The progestogen, DHP levels were positively and significantly correlated with salinity in males. Most of our knowledge of DHP in ectotherms are from studies of fish where it acts as an oocyte maturation inducing hormone in female teleosts^61,62^ and in maturation of sperm and induction of sperm motility in males^63,64^. To the best of our knowledge, this study presents the first measurement of DHP in alligators. It is currently unknown what role DHP plays in juvenile alligators exposed to salinity. It is interesting to note, however, that DHP is involved in reproduction in fish, so therefore could potentially play a similar role in egg-laying reptiles. As there is evidence from laboratory studies that salinity levels affect reproduction (changes in gonad histology in males and females) and affects key sex steroid hormone levels, the significant correlation between DHP and salinity in males provides further evidence of potential reproductive disruptions in alligators exposed to salinity.

Several steroid hormones varied by month in wild male juvenile alligators, including an increase in testosterone (not significant) during May and peaking in June, which corresponded with similar trends of increased androstenedione and 5α-dihydrotestosterone. Increased testosterone around March and April is followed by the onset of mating season which normally starts in April^35,65^. Indeed, previous studies have demonstrated cycling of testosterone in wild adult and juvenile alligators during the months of March, April, and May^60,66–68^. However, the present study only detected increased male androgens in May and June. Testosterone peaks in June correspond well with increased testosterone previously reported in 90–119 cm males^68^ and in other juvenile males^60^. However, the peak testosterone levels presented in this study (25.74 ng ml^−1^) are closer to those of adult males less than 135 cm snout-vent length (~ 25 ng ml^−1^)^66^ than to wild juvenile alligators between 56–172 cm^60^ and 59–180 cm total length^68^ (below 8 ng ml^−1^). The variable levels of sex hormones seen in reptiles can be due to various environmental factors. For instance, seasonal variation in lizard sex hormones can be influenced by temperature^69,70^ or diet^71,72^. Further study is required to understand the factors controlling the seasonal fluctuation in juvenile alligators across size classes and whether salinity levels can impact this seasonal variation in sex steroid hormone levels.

Although only one female alligator was opportunistically sampled during May, the higher (not significant) 17β-estradiol in this sample compared with females sampled during other months corresponds well with the higher level of its substrate testosterone, as well as androstenedione and 5α-dihydrotestosterone. Seasonal variation in 17β-estradiol has previously been reported in other alligator populations where adult^58,59^ and juvenile^60^ female alligators in Florida exhibited increases in 17β-estradiol during the months of March, April, and May. Smaller, subsequent increases in sex steroid hormones can also occur, such as 17β-estradiol in August, September, and October^58–60^ or in testosterone during August^60^. Furthermore, estrone levels in juvenile females, in the current study, also tended to increase in April, similarly coinciding with mating season. On the other hand, estriol levels remained low during April and May, but tended to be higher during June, correlating with alligator nesting season. While not significant, progesterone and 17α-hydroxyprogesterone tended to increase in juvenile females during May and June, similar to trends seen in progesterone levels of adult females sampled in Florida lakes^58^. However, adult female alligators sampled on a barrier island have shown more stable progesterone levels across seasons^59^, illustrating differences between some alligator populations. Overall, sex steroid levels in immature alligators appear to reflect similar seasonal variation as seen in adults. Furthermore, the fluctuations in sex steroids reported in the present study occur independently from varying environmental salinity since hyperosmotic samples were only obtained in August, September, and October 2018. Further work is needed however to understand the role these fluctuations play in the maturation of juvenile alligators, and if changes in these hormone levels resulting from environmental changes (e.g., salt inundation) impact the long-term fitness of juvenile alligators.

The present study demonstrates significant correlations between exposure to hyperosmotic water and blood plasma electrolyte concentrations (Na^+^, Cl^−^) in wild juvenile alligators, similar to changes seen in laboratory-kept juvenile alligators exposed to 12‰ seawater. In addition, higher environmental salinity was associated with elevated levels of the progestogen DHP in juvenile male alligators. Glucocorticoid, androgen, and estrogen levels were not clearly impacted in hyperosmotic salinities, likely due to confounding factors present in the wild and differences in salinity exposure time compared with laboratory-based studies. Further, the RAAS hormones angiotensin II and aldosterone were not significantly correlated with high salinity levels in males, but aldosterone was in fact significantly correlated with hypoosmotic environments in juvenile female alligators.

These findings evoke questions regarding the physiological changes in alligators exposed to hyperosmotic environments. Further research is needed to understand what role DHP plays in alligators. Future studies should also investigate if exposure to high salinity during developmental stages of juvenile alligators impacts long-term fitness, and to observe whether salt inundation affects wild male alligators differently than females due to territorial social interactions and differential levels of sex steroid hormones. This information will be crucial in understanding how wild alligator populations in vulnerable Gulf of Mexico ecosystems respond to natural stressors such as salinity in a changing climate.

## Data Availability

All data generated or analyzed during this study are included in this article.
